# Towards a Better Understanding of the Human Health Risk of Per- and Polyfluoroalkyl Substances Using Organoid Models

**DOI:** 10.3390/bioengineering12040393

**Published:** 2025-04-07

**Authors:** Haoan Xu, Jiahui Kang, Xue Gao, Yingying Lan, Minghui Li

**Affiliations:** 1 School of Life Sciences and Technology, Tongji University, Shanghai 200120, China; haoanxu2023@163.com; 2 Key Lab of Visual Damage and Regeneration & Restoration of Chongqing, Southwest Hospital, Third Military Medical University (Army Medical University), Chongqing 400038, China; 18723150911@163.com; 3 Key Laboratory of Biorheological Science and Technology, Ministry of Education, College of Bioengineering, Chongqing University, Chongqing 400030, China; gaoxx3020@163.com; 4 Institute of Burn Research, Southwest Hospital, Third Military Medical University (Army Medical University), Chongqing 400038, China

**Keywords:** per- and polyfluoroalkyl substances, PFAS, human health, human organoids, toxicity assessment

## Abstract

The ubiquitous presence of per- and polyfluoroalkyl substances (PFAS) in the environment has garnered global public concern. Epidemiological studies have proved that exposure to PFAS is associated with human health risks. Although evidence demonstrated the toxic mechanisms of PFAS based on animal models and traditional cell cultures, their limitations in inter-species differences and lack of human-relevant microenvironments hinder the understanding of health risks from PFAS exposure. There is an increasing necessity to explore alternative methodologies that can effectively evaluate human health risks. Human organoids derived from stem cells accurately mimic the sophisticated and multicellular structures of native human organs, providing promising models for toxicology research. Advanced organoids combined with innovative technologies are expected to improve understanding of the breadth and depth of PFAS toxicity.

## 1. Introduction

Per- and polyfluoroalkyl substances (PFAS) are a group of more than 10,000 fluorinated synthetic chemicals whose structures contain at least one fully fluorinated methyl or methylene carbon atom. PFAS are often referred to as “forever chemicals” because of their innate chemical stability [1]. PFAS are ubiquitously present in natural environments due to their widespread use and persistent nature, resulting in pollution problems of unprecedented scale [2]. PFAS have been detected in rivers, soil, air, house dust, and the food supply [3,4]. Perfluorooctanoic acid (PFOA) and perfluorooctane sulfonate (PFOS) are two of the most used PFAS in various applications. Although they have been phased out, materials containing PFOA and PFOS are still used in consumer and industrial products. Thus, humans are continuously exposed to them. In response, an array of emerging PFAS have been used to replace PFOS and PFOA and are also increasingly detected in environmental and human samples [1,5,6,7,8].

Given ubiquitous exposure and long half-lives, it is critical to understand the adverse effects of PFAS. Traditional animal and cell line models are commonly used to identify and predict potential adverse effects of PFAS on humans. However, animal models did not accurately predict the toxic responses in humans [9,10]. Moreover, using experimental animals is limited by ethical issues and does not comply with the 3R principles (replacement, reduction, and refinement of animal studies) [11]. Traditional two-dimensional (2D) cell assays cannot mimic the human-relevant physiological microenvironments [12]. Three-dimensional (3D) organoid models represent new in vitro models that will complement existing cell lines and animal models to a certain extent [13,14,15]. Organoid toxic tests are expected to be novel alternative methods in environmental toxicology [16,17,18].

## 2. PFAS Pose Threats to Human Health

**Figure 1:**
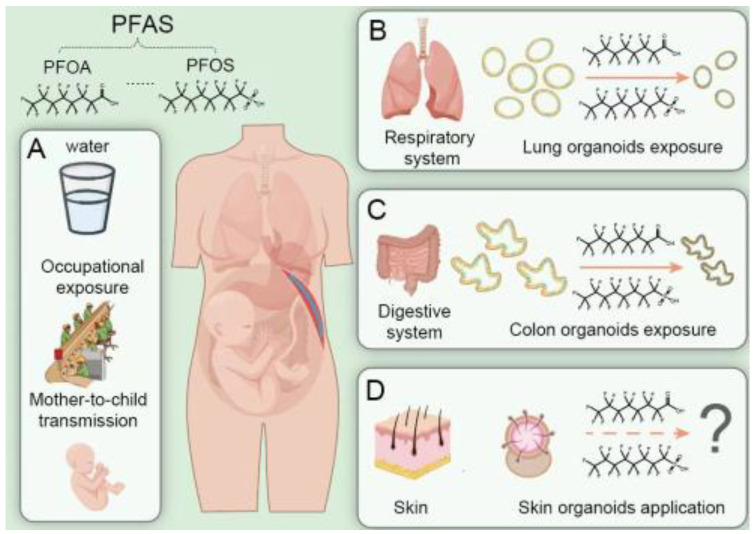
PFAS exposure routes and organoid models for toxicity assessment. (**A**) Drinking water and occupational exposure are the main routes of PFAS exposure. PFAS can be transmitted from mother to fetus through the placenta. (**B**–**D**) Human organoid models can be applied to evaluate the toxicity of PFAS.

The primary routes of human exposure to PFAS include contaminated water, food, air, and consumer and personal care products [19,20]. Drinking water is the most significant source of human exposure to PFAS (Figure 1) [21]. Scientists estimated that over 200 million people in the United States are served by PFAS-contaminated water at a concentration of 1 part per trillion [22]. The data from a nationwide prospective cohort of United States women in the Nurse’s Health Study demonstrated that tap water contributes to plasma concentrations of PFAS [23]. Occupational exposure to PFAS can be another source of fluorochemical workers, firefighters, and electronic waste workers [24,25,26,27]. Increasing epidemiological studies have demonstrated that PFAS exposure is positively associated with adverse human health outcomes, including cancers (e.g., testicular, kidney, prostate, bladder, breast, and ovarian cancers), immune disruption, liver damage, adverse reproductive effects, and developmental delays [4,23,28]. For instance, serum PFOA levels were positively related to renal cell carcinoma [29,30,31]. Although PFOA exposure was found to induce histological and cellular changes in renal tubules in animal models [31,32], its underlying mechanisms remain unclear.

Since PFAS have been ubiquitously detected in human blood, a strong positive association between serum PFAS concentrations and cardiovascular risk has been widely identified [25]. A previous study showed that PFOA-induced cardiac malformations and dysfunction could be mediated by oxidative stress, which was caused by activating the Keap1/Nrf2 pathway [33]. Moreover, studies have proved that PFAS can penetrate the blood-placental barrier and enter the fetus from the maternal side, posing threats to fetal development [34,35,36]. Prenatal exposure to PFOA impaired the competence of maturing oocytes and reduced the yield of oocytes in the progeny mice [37]. However, human response to PFAS exposure has not been clearly defined because of insufficient epidemiological data and human-relevant models.

## 3. Potential of Human Organoids in PFAS Toxicity Assessment

Organoid is a self-organized 3D tissue that can be derived from stem cells and tissue cells, which simulate the key structure, function, and biological complexity of the native organ [38,39]. Since the first in vitro 3D intestinal organoid was established by Clevers’ lab in 2009, organoid technology has rapidly advanced over the past decade [39,40]. To date, various organoids, such as brain, retinal, kidney, cardiac, airway, and liver organoids, have been successfully established. Existing evidence has emphasized the importance of human organoids in modeling diseases, drug discovery, and precision medicine [41,42]. More recently, the application of human organoids has greatly expanded the scope of predictive toxicology research [10].

Human exposure to environmental toxicants occurs mainly through ingestion, inhalation, and dermal absorption, so human organoids of corresponding systems are employed for toxicity assessment (Figure 1). For instance, lung organoids were established to reveal the impacts of diesel exhaust particles (DEP) on the alveolar progenitor niche [43]. Human lung bud tip progenitor organoids were also used to evaluate the elemental toxicity of particulate matter (PM2.5) [44]. PM2.5-induced effects on the proliferation and differentiation of lung organoids partially involved the Wnt/β-catenin signaling pathway. Given that previous research has proved the lung toxicity of PFAS, lung organoids are expected to reveal the potential toxicity of PFAS to the human lungs and their toxicity mechanisms [45,46,47].

The primary pathway for human exposure to PFAS is the consumption of contaminated food and water [48]. The existing gastrointestinal organoids, such as intestinal, colon, and stomach organoids, can mimic the structure and function of the intestinal epithelium, enabling the capture of toxicant-induced gastrointestinal toxicities [49,50]. It was reported that PFOS exposure influenced the absorption function of the intestinal epithelium in intestinal organoids [48]. PFOS-induced absorption of fatty acids and formation of lipid droplets were mediated through the regulation of the PPARα pathway. The effect of PFOS on activating the PPARα pathway of intestinal organoids was stronger than that of PFOA [48].

The skin is the largest human organ and is continuously exposed to environmental toxicants. The phasing out of animal testing for cosmetic products has facilitated the development of human organoid models. A cyst-like skin organoid comprising stratified epidermis, fat-rich dermis, pigmented hair follicles, and sebaceous glands has been generated, equivalent to human fetuses’ facial skin [51]. Dermal absorption is supposed to be an important pathway of human exposure to PFAS [52]. Hence, skin organoids hold great potential for assessing PFAS toxicity in the future.

Enormous organoids have been used to decipher the possible neurotoxicity, hepatoxicity and kidney toxicity of compounds due to their transport, accumulation, and biotransformation in the human body. For instance, brain organoids were employed to illustrate the potential neurotoxicity of the PFAS mixture (PFOS, PFOA, and PFHxS). PFAS-exposed brain organoids exhibited amyloid beta accumulation and tau phosphorylation, which were Alzheimer’s Disease (AD)-like phenotypes [53]. Consistently, developmental PFOS exposure was identified as a potential risk factor for late-onset AD in CD-1 mice [54]. Treatments of liver organoids with short-chain PFAS induced the loss of architectural complexity and aberrant cytological features [55]. Given that exposure to PFAS poses threats to the human reproductive system, ovarian, trophoblast, and placental organoids might help to illustrate their potential reproductive toxicity [56,57,58,59].

## 4. Advances and Challenges of Human Organoids

**Figure 2:**
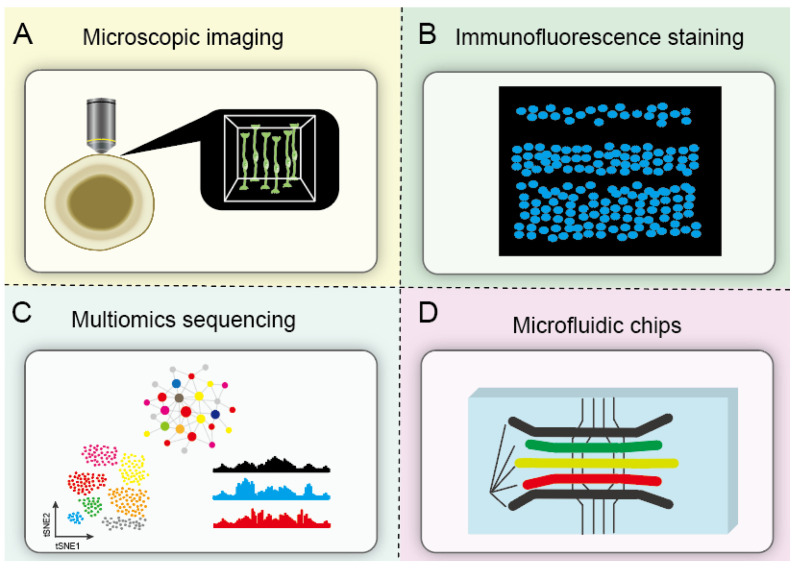
Advances and challenges in human organoid models. (**A**) Observing human organoids by microscopy is a simple and convenient method for quantitative analysis. (**B**) Immunofluorescence staining revealed biochemical changes in organoids after exposure to toxicants. (**C**) Multi-omics sequencing can further explore toxic mechanisms. (**D**) Microfluidic chips can reduce the heterogeneity of organoid culture and further improve the existing organoid culture methods.

Human organoids share similar microstructure and morphology with their source organs/tissues; observing the morphological changes and growth characteristics by a microscope is a simple and convenient method to determine the toxic effects of toxins (Figure 2). For example, the decreased area and thickness of retina epithelial structures were found after retinal organoid exposure to polybrominated diphenyl ethers (PBDEs) [60]. Fluorescence imaging from immunofluorescent staining also offers a specific visualization option that will allow for identifying cell types and their arrangement in proper layers within organoids. The quantification of specific cell markers and live/death assay enables tracking organoid growth and development upon pollutant exposure. Di-(2-ethylhexyl) phthalate was found to inhibit cell proliferation, induce apoptosis, and disrupt cell migration in cortical organoids [61].

The “omics” technologies, transcriptomics, proteomics, epigenomics, and metabolomics, have been employed to explain or predict the toxic mechanisms. Omics-based approaches offer valuable insight into potential biomarkers of organoid responses under stress (Figure 2). For instance, RNA sequencing was applied to illustrate the underlying molecular mechanisms of bisphenol-induced retinal toxicity in retinal organoids [10]. Bisphenol treatment also induced distinct proteomic changes in mammary gland organoids [62]. Metabolomics has been employed to uncover the active metabolic pathways involved in fatty acid metabolism, nucleotide metabolism, necroptosis, and autophagy pathways under nanoparticle stress in intestinal organoids [63]. Currently, integration strategies of multi-omics data are used to obtain a more comprehensive understanding of the toxicological mechanisms of environmental pollutants. The integrated networks of altered metabolites and differentially expressed genes revealed that PBDE-induced retinal toxicity was closely associated with glutathione metabolism and oxidative stress [60]. Recent technological advances in single-cell manipulation offer unprecedented opportunities for profiling genes and proteins at a single-cell resolution. Unlike conventional omics approaches, cutting-edge single-cell omics profiling technologies provide novel insight into the mechanisms of responses to toxicants of individual cells within organoids [64]. Single-cell RNA sequencing data of intestinal organoids revealed the adverse impact of bisphenol A and its substitute, fluorene-9-bisphenol, on intestinal homeostasis [65]. The differentiation trajectory of the intestinal cells was significantly influenced after chemical exposure. The emergence of spatial multi-omics has promoted the development of single-cell sequencing [66]. Deciphering the spatiotemporal omics profile of organoids at the single-cell level will accelerate the development of toxicity assessment and advance the understanding of the toxic mechanisms of PFAS [67].

Notably, traditional organoid models suffer from considerable variability and heterogeneity [68]. Engineering strategies based on advanced technologies have been applied to increase organoid reproducibility. For instance, biomaterials, poly(ethylene glycol) (PEG)-based hydrogels, can be designed to promote reproducible intestinal organoid formation [69]. Furthermore, the potential of organoid technology was hindered by the low-level maturity and functions due to the lack of stromal, vascular, and immunological components [38]. Great progress has been made in enhancing vascularization and immunization by incorporating organoids with endothelial and immune cells [70,71,72]. Critically, organ crosstalk and communications between different cell types play a fundamental role in maintaining whole-body homeostasis; individual organoids cannot construct a multi-organ system in vivo. Organs-on-a-chip, engineered or natural miniature tissues grown inside microfluidic chips, provide microphysiological environments for organoid systems [73,74]. Cardiac organoid-on-a-chip and brain organoid-on-a-chip have been applied to investigate polystyrene nanoplastic-induced cardiotoxicity and nicotine-induced neurotoxicity, respectively [75,76]. Such capabilities have recently been demonstrated by an integrated multi-organoid platform comprising the liver, cardiac, lung, vascular, testis, colon, and brain organoids [77]. The multi-organoid system shows great advantages in modeling the metabolism and downstream effects of drugs between multiple tissues [77,78,79]. Human organoids-on-a-chip will be performed to reveal the systematic toxicity of PFAS in the near future.

## 5. Conclusions

Despite human organoids promoting the development of toxicology, the application of organoids in PFAS toxicity assessment is still in its infancy. One aspect is that PFAS exerts persistently toxic effects, but the high cost inhibits their long-term observation. Another aspect is that humans are simultaneously exposed to multiple PFAS rather than individual chemicals. Thus, organoids combined with state-of-the-art technologies and advanced calculation methods are expected to be developed to illustrate the toxicity of PFAS. Recent organoid-on-a-chip microfluidic devices have enabled high-throughput toxicity assessment, thus promoting the understanding of the potential effects of all PFAS species on human health [78,80]. Moreover, the organoid-on-a-chip system offers the possibility to study the biotransformation and metabolism of labile PFAS in human organs, which cannot be mimicked in traditional cell cultures and animal models [73,74,79,81]. Organoid-on-a-chip also offers advantages over traditional 2D cultures in maintaining the interactions between cells and organs, simulating human-relevant physiological conditions, and greater cellular diversity [16,82].

All in all, human organoid models provide a promising platform to determine the potential toxicity of PFAS and mechanisms of action. Evidence from epidemiological studies will benefit in verifying the relationship between human health and PFAS exposure. Once deleterious effects are identified, adverse health outcomes are not easily reversed due to the technical challenge. Thus, preventative upstream actions are critical to minimizing the impact of PFAS on human health.

## Data Availability

No data were used in this study.
